# 应用MALDI-TOF-MS检测肺鳞癌患者血清多肽并分析其与化疗疗效相关性

**DOI:** 10.3779/j.issn.1009-3419.2017.05.04

**Published:** 2017-05-20

**Authors:** 冠华 赵, 斌 许, 晓燕 李, 传昊 汤, 海峰 秦, 红 王, 绍兴 杨, 伟霞 王, 红军 高, 昆 何, 晓晴 刘

**Affiliations:** 1 100071 北京，军事医学科学院附属医院肺部肿瘤科 Department of Lung Cancer, Affiliated Hospital of Academy of Military Medical Sciences, Beijing 100071, China; 2 100850 北京，国家生物医学分析中心 National Center of Biomedical Analysis, Beijing 100850, China

**Keywords:** 肺肿瘤, 化疗, 基质辅助激光解析电离飞行时间质谱, 蛋白质组学, 治疗疗效, Lung neoplasms, Chemotherapy, Matrix-assisted laser desorption/ionization time-of-flight mass spectrometry, Proteomics, Therapeutic effect

## Abstract

**背景与目的:**

晚期肺鳞癌（squamous cell carcinoma of lung, SCC）一线治疗以化疗为主，其标准铂二联方案化疗只能给患者带来有限的获益。并且不同的患者对于化疗药物的获益不同。所以实现化疗药物最优选择达到个体化预见性治疗尤为重要。本研究应用基质辅助激光解析电离飞行时间质谱（matrix-assisted laser desorption/ionization-time of flight-mass spectrometry, MALDI-TOF-MS）检测初治晚期SCC患者接受紫杉醇类联合铂类化疗前血清多肽，并分析其与化疗疗效的相关性。

**方法:**

初治晚期SCC患者接受紫杉醇类联合铂类方案化疗，每两周期进行疗效评价。评效为完全缓解（complete response, CR）或部分缓解（partial response, PR）患者定义为化疗敏感组，疾病进展（progressive disease, PD）患者定义为耐药组。留取SCC患者化疗前血清样本，81例患者按照3:1的比例随机分为训练组（敏感组Ⅰ与耐药组Ⅰ）和验证组（敏感组Ⅱ与耐药组Ⅱ），预处理训练组血清样本并进行MALDI-TOFMS检测，得到血清多肽指纹图谱。经ClinProTools软件系统分析处理，得到敏感组Ⅰ与耐药组Ⅰ的差异多肽。应用软件内置的3种不同的生物学算法分别建立疗效预测模型，选取最优算法建立疗效预测模型。运用验证组进行盲样验证。

**结果:**

训练组共纳入30例敏感组患者，31例耐药组患者；验证组共纳入敏感与耐药组患者各10例。训练组在敏感与耐药组有96个差异多肽，其中具有统计学意义的多肽有16个（*P* < 0.001）。由5个多肽（1, 897.75 Da, 2, 023.93 Da, 3, 683.36 Da, 4, 269.56 Da, 5, 341.29 Da）建立疗效预测模型。该模型对化疗敏感组患者的识别率为95.11%，交叉验证率为89.18%。经验证组进行盲样验证，其模型的准确率为85%，灵敏度为90.0%，特异性为80.0%。敏感组Ⅰ中位无进展生存期（progress free survival, PFS）为7.2个月(95%CI: 4.4-14.5)；耐药组Ⅰ中位PFS为1.8个月（95%CI: 0.7-3. 5）。结果发现：4, 232.04 Da、4, 269.56 Da的差异多肽与SCC患者PFS存在相关性（*P* < 0.001）。

**结论:**

应用MALDI-TOF-MS技术可检测到化疗敏感组及耐药组患者的血清多肽存在差异，初步建立的疗效预测模型可用于预测紫杉醇类联合铂类方案化疗疗效。但需进一步扩大样本量完善及验证模型。

## Abstract (en)

**Background and objective:**

Treatment options for patients with squamous cell carcinoma of the lung (SCC) are limited in chemotherapy. However, not all patients could benefit form standard platinum regimen. Considering the dismal prognosis of patients with advanced SCC, a greater focus on selecting sensitive chemotherapy regimens remains of upmost importance to improve outcomes in this disease. In this study, we used matrix-assisted laser desorption/ionization timeof-flight mass spectrometry to detect pre-chemotherapy serum peptides in advanced lung squamous cell carcinoma patients accepting paclitaxel combined with platinum chemotherapy and to analyze the correlation between serum peptides and chemotherapy efficacy.

**Methods:**

Patients with advanced lung squamous cell carcinoma received paclitaxel combining with platinum chemotherapy and evaluated the efficacy every two cycles. Evaluation of complete response (CR) or partial response (PR) patients defined as sensitive group, progressive disease (PD) patients defined as resistant group. Serum samples were collected from patients with lung squamous cell carcinoma. Eighty-one patients were randomly divided into training group (sensitive group Ⅰ and resistant group Ⅰ) and validation group (sensitive group Ⅱ and resistant group Ⅱ) according to the ratio of 3:1. Serum samples were pretreated and Matrix-assisted laser desorption/ionization time-of-flight mass spectrometry (MALDI-TOFMS) was used to detect serum peptide fingerprints. ClinProTools software was used to analyze the differences between the sensitive group Ⅰ and the resistant group Ⅰ. Three kinds of biological algorithms (SNN, GA, QC) built in CPT software were used to establish the curative effect prediction model respectively and the optimal algorithm was selected. The validation group was used for blind verification.

**Results:**

Thirty sensitive patients and 31 resistant patients were enrolled in the training group. Ten sensitive patients and 10 resistant patients were included in the validation group. The training group had 96 differentially expressed peptides in the sensitive and resistant patients, with 16 statistically significant peptides (*P* < 0.001). The predictive model was established by 5 polypeptides (1, 897.75 Da, 2, 023.93 Da, 3, 683.36 Da, 4, 269.56 Da, 5, 341.29 Da). The recognition rate of this model was 89.18% and the cross validation rate was 95.11%. The accuracy of the model was 85%, the sensitivity was 90.0% and the specificity was 80.0%. The median PFS in the sensitive group was better than patients in the resistant group (7.2 months 95%CI: 4.4-14.5 *vs* 1.8 months 95%CI: 0.7-3.5). The results showed that the differential peptides 4, 232.04 Da and 4, 269.56 Da were correlated with PFS in patients with lung squamous cell carcinoma (*P* < 0.001).

**Conclusion:**

MALDI-TOFMS was used to detect the difference of serum peptides between sensitive and resistant groups. The preliminary curative effect prediction model was used to predict the efficacy of paclitaxel combined with platinum regimen. However, this model need further investigations to verify the accuracy and the sensitivity.

肺癌（lung cancer）目前是临床上最常见的恶性肿瘤。其中80%为非小细胞肺癌（non-small cell lung cancer, NSCLC），最常见的病理类型包括：肺鳞癌（squamous cell carcinoma of lung, SCC）与肺腺癌（adenocarcinoma of lung）^[1, 2]^。尽管抗血管生成治疗、免疫治疗、靶向治疗等已被食品药品监督管理局（Food and Drug Administration, FDA）批准为晚期SCC患者的二线或多线治疗手段。晚期SCC的临床治疗仍停留在以传统化疗为主的阶段，铂二联方案化疗依然是晚期SCC患者主要的一线治疗手段^[3]^。但是对于不同SCC患者，其化疗疗效却不相同。近年来虽然进行了多项针对单基因标志物如ERCC1、RRM1、TUBB3及XRCC1的研究，并初步显示出一定意义，但在进一步扩大样本验证的随机对照Ⅲ期临床研究中均未能有效预测疗效^[4, 5]^。所以迄今为止无一标志物可用于指导临床化疗药物的选择。因此临床上亟需一种可以更有效预测SCC化疗疗效的方法。质谱检测蛋白质组学研究已广泛应用于肿瘤学的各个领域^[6-14]^，既往研究发现化疗药物耐药性及药物代谢酶多态性可导致不同的患者对于化疗药物的获益不同。因此本研究应用MALDI-TOF-MS技术从蛋白质及多肽等更微观的角度了解初治晚期SCC患者化疗前血清多肽与化疗疗效的关系，期望为实现晚期SCC患者的个体化化疗奠定基础。

## 1 材料与方法

### 1.1 样本

本研究共入组81例2014年10月-2016年4月期间就诊于解放军第307医院肺部肿瘤内科的初治晚期SCC患者，在患者未进行任何治疗时采集血清样本，告知并签署知情同意书。纳入患者满足以下标准：①经组织病理学或细胞学确诊为SCC的患者（Ⅲb期、Ⅳ期）；②年龄≥18周岁；③美国东部肿瘤协作组体力评分（Eastern Cooperative Oncology Group Performance Status, ECOG PS） < 2分；④入组患者均排除心、肝、肾等重要脏器疾病；⑤此前未接受过化疗、放疗、靶向治疗等肿瘤专科治疗；⑥一线治疗行紫杉醇类联合铂类方案化疗，紫杉醇（135 mg/m^2^-175 mg/m^2^）+顺铂（75 mg/m^2^）或卡铂[峰下面积（area under curve, AUC）=5-6）]，多西他赛（60 mg/m^2^-75 mg/m^2^）+顺铂（75 mg/m^2^）或卡铂（AUC =5-6），第1天用药（顺铂第1、2天给药），每3周重复。

### 1.2 试剂和仪器

三氟乙酸（Trifluoracetic acid，TFA，美国Sigma公司）；乙腈（Acetonitrifle，CAN，美国Fisher公司）；α-氰基-4-羟基肉桂酸（α-cyano-4-hydroxycinnamic acid, HCCA）和混合标肽（Bruker公司）；铜离子螯合纳米磁珠（MB-IMAC-Cu^2+^）；缓冲液体系（国家生物医学分析中心）；MALDI-TOF质谱仪（Ultraflex）（德国Bruker公司）；磁珠分离器（Magnetic bead separator; MBS）（德国Bruker公司）；SIGMA台式冷冻离心机3K15（美国SIGMA公司）；MTP-Anchorpchip靶（Var/384）（德国Bruker公司）；分析软件ClinProTools 3.0版（CPT, 德国Bruker公司）。

### 1.3 方法

#### 1.3.1 患者分组

纳入的初治晚期SCC患者，一线行紫杉醇类联合铂类方案化疗，并每两周期进行疗效评价，记录患者近期疗效、无进展生存期（progression free survival, PFS），从接受化疗开始，到评价疾病进展或者发生因为任何原因的死亡为止）。按照实体瘤疗效评价标准（Response Evaluation Criteria in Solid Tumors, RECIST）1.1评价治疗疗效。化疗疗效评价为完全缓解（complete response, CR），部分缓解（partial response, PR），疾病稳定（stable disease, SD），疾病进展（progressive disease, PD）。本研究仅选取CR、PR、PD的患者入组。将评效为CR或PR的SCC患者定义为化疗敏感组，评效为PD的SCC患者定义为化疗耐药组。将入组标本按照3：1的比例随机分为训练组（敏感组Ⅰ与耐药组Ⅰ）和验证组（敏感组Ⅱ与耐药组Ⅱ）。

#### 1.3.2 血清样本采集

化疗前晨起空腹取外周血3 mL，尿液10 mL。4 ℃冰箱静置30 min后，4 ℃低温离心：4, 000 rpm、10 min，取上层血清分装于冻存管，于-80 ℃冰箱保存。

#### 1.3.3 血清预处理

从4 ℃冰箱中取出SPE-CM磁珠悬浮液一管，反复倒置，使磁珠完全、均匀的悬浮与液相中。在200 μL样品管中加入7 μL SPE-CM磁珠混悬液，10 μL血清、95 μL SPE-CB反复吸打数次，使磁珠与SPE-CB、血清混合均匀，室温静置5min。将样品管置于磁珠分离器上，磁珠贴壁1 min，使磁珠与液体分离，待液体澄清后，吸净液体。将样品管从磁珠分离器上移走，并加入100 μL SPE-CW，反复吸打数次，使磁珠与SPE-CW混合均匀，室温静置2 min。将样品管转移到磁珠分离器上，磁珠贴壁1 min，使磁珠与液体分离，待液体澄清后，吸净液体，重复上步2次。将样品管中加入10 μL SPE-CE，反复吸打10次以上，使磁珠与SPE-CE混合均匀，室温静置5 min。将样品管转移到磁珠分离器上，磁珠贴壁1 min，使磁珠与液体分离，待液体清澈后，将液体移入干净的样品管中。冻存于-20 ℃冰箱，待进行质谱分析。

#### 1.3.4 点靶

将饱和的HCCA于0.1% TFA和50% ACN的混合液混匀于Eppendorf管内，配成基质溶液，将基质溶液与标准品按照2:1的比例均匀混合，吸取1 μL点样，外部校正仪器。再将磁珠处理后的待检样本1 μL与预配基质溶液1 μL按照1:1比例均匀混合，吸取1 μL点于MTP-Anchorpchip靶上，室温静置等待自然干燥。

#### 1.3.5 质谱检测

将点靶完成的MTP-Anchorpchip靶放入MALDI-TOF-MS进行分析。为降低操作误差和系统误差，每组样本检测前均用1个标准品（标准的多肽混合物）点靶，作外标进行检测，外部校正仪器。MALDITOF-MS最佳扫描的质荷比（mass-to-charge ratio, m/z）区间为800 Da-10, 000 Da，质谱信号每次扫描累加500次，每份样本共累加3, 000次，保存累加图谱，从而得到精准的血清多肽指纹图谱，图谱由各个不同m/z的多肽峰组成。

### 1.4 统计学分析

MALDI-TOF-MS分析数据经CPT软件进行质谱图谱处理，分别得到SCC患者的血清指纹多肽质谱。比较敏感组Ⅰ与耐药组Ⅰ的多肽指纹图谱，用软件内置的3种算法：快速分类法（quickclassifier, QC算法）、遗传算法（genetic algorithm, GA算法）和监督神经网络算法（supervised neural network, SNN算法），分别建立疗效预测模型。选出其中最优算法所建立的疗效预测模型，并自动生成了建模所用多肽并计算了多肽AUC。随后应用验证组样本对所建模型进行盲样验证。运用SPSS软件（版本windows 19.0）对入组训练组和验证组的临床特征：年龄、性别、吸烟进行配对四个表卡方检验，用以分析两组人群临床特征的差异。将敏感组Ⅰ及耐药组Ⅰ的差异多肽结合临床预后参数PFS运用双变量相关分析，得到各个差异多肽与PFS间的相关系数，即*pearson*相关系数。检测血清差异多肽与SCC患者PFS的相关性。

## 2 结果

### 2.1 入组患者一般临床特征

共入组81例患者，训练组及验证组在年龄、性别、吸烟情况及临床分期等方面无统计学差异，入组患者具体信息见表 1。

**1:** 患者基本情况 Basic information of patients

Characteristic	Training group (*n*=62)		*P*	Validation group (*n*=20)		*P*
	Good (*n*=10)	Bad (*n*=10)		Good (*n*=10)	Bad (*n*=10)	
Age (yr)			0.459			0.421
Median	63	62		64	60	
Range	42-75	44-77		45-80	37-74	
Gender			0.722			0.531
Male	27 (90%)	27 (87%)		8 (80%)	9 (90%)	
Female	3 (10%)	4 (13%)		2 (20%)	1 (10%)	
Smoking			0.542			0.606
Yes	22 (73%)	24 (77%)		7 (70%)	8 (80%)	
No	8 (27%)	6 (23%)		3 (30%)	2 (20%)	
Staging						
Ⅲ	3 (11%)	4 (13%)		1 (10%)	1 (10%)	
Ⅳ	27 (89%)	27 (87%)		9 (90%)	9 (90%)	

### 2.2 疗效分析

81例接受紫杉醇类联合铂类化疗的初治晚期SCC患者随访至2016年12月，全部患者均进展。其中一线评效，CR 0例，PR 40例（40/81, 49.4%），PD 41例（41/81, 50.6%）。训练组共纳入30例敏感患者（敏感组Ⅰ），31例耐药患者（耐药组Ⅰ）；验证组共纳入敏感（敏感组Ⅱ）与耐药（耐药组Ⅱ）患者各10例。敏感组Ⅰ中位PFS为7.2个月（95%CI: 4.4-14.5）；耐药组Ⅰ中位PFS为1.8个月（95%CI: 0.7-3.5）。

### 2.3 训练组的血清多肽指纹图谱

对训练组血清样本进行预处理，MALDI-TOF-MS质谱检测，得到敏感组Ⅰ及耐药组Ⅰ的血清多肽指纹图谱，结果见图 1、图 2。

**1:**
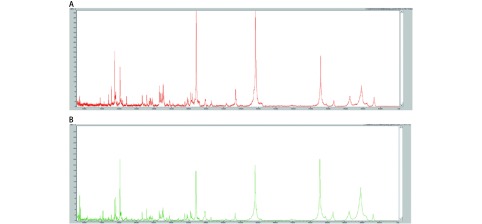
训练组的血清多肽指纹图谱（A：敏感组Ⅰ；B：耐药组Ⅰ） Serum peptide profiles of training group (A: sensitive groupI; B: resistant group Ⅰ)

**2:**
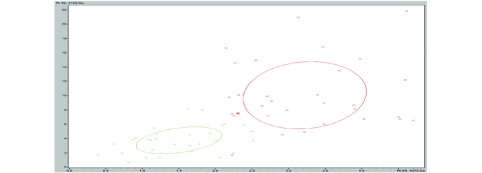
血清多肽图的聚类分析（红色：敏感组Ⅰ；绿色：耐药组Ⅰ） Clustering analysis of MSbased serum peptide profiles (red: sensitive group Ⅰ; green: resistant group Ⅰ)

### 2.4 训练组的血清差异多肽结果分析

#### 2.4.1 化疗疗效相关多肽的发现

运用CPT软件对敏感组Ⅰ及耐药组Ⅰ的血清指纹图谱进行处理，在800 Da-10, 000 Da范围内，检测到96个差异多肽，统计学处理后具有统计学意义（*P* < 0.001）的差异多肽有16个，具体信息见表 2。

**2:** 训练组差异多肽质荷比及表达变化 m/z and expression change of training group

m/z	Good	Bad	Expression levels of the bad compared to the good
4, 269.56	3.58±0.94	1.67±0.65	↓
3, 159.48	11.16±5.23	4.16±2.09	↓
4, 284.47	4.12±2.04	1.5±0.57	↓
4, 212.4	36.88±12.09	18.55±11.21	↓
4, 093.33	5.96±1.97	3.19±1.64	↓
3, 957.44	5.57±1.83	3.02±1.7	↓
3, 974.86	4.16±1.84	2.06±0.89	↓
3, 683.36	1.52±0.47	1.03±0.32	↓
2, 023.93	16.39±13.7	23.15±25.32	↑
4, 232.04	6.22±2.03	3.86±2	↓
3, 193.45	4.2±1.27	2.69±1.38	↓
3, 181.56	2.91±1.03	1.88±0.8	↓
1, 897.75	4.75±2.81	9.39±3.72	↑
4, 646.27	3.59±2.47	1.58±0.68	↓
4, 300.74	3.07±1.64	1.72±0.79	↓
5, 341.29	4.49±2.33	2.02±2.2	↓

#### 2.4.2 化疗疗效预测模型的建立

运用CPT软件将最具统计学差异的两个峰（3, 159 Da, 4, 270 Da）分别作为横纵坐标绘制了2D多肽峰聚类分析图（图 3）。用软件内置的3种算法（SNN、GA、QC算法）分别建立诊断模型，选出其中最优算法（GA算法）所建立的诊断模型。运用GA算法建立的化疗疗效预测模型，由5个多肽组成，分别为1, 897.75 Da、2, 023.93 Da、3, 683.36 Da、4, 269.56 Da、5, 341.29 Da（图 4）。该模型对化疗敏感组患者的识别率为95.11%，交叉验证率为89.18%。

**3:**
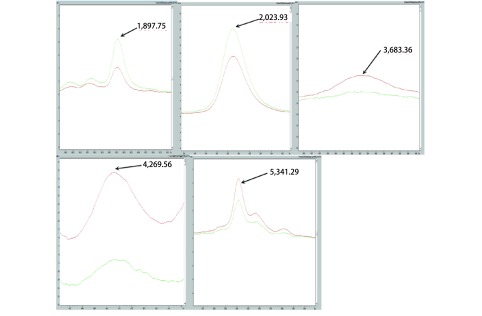
建模的5个多肽峰（红色：敏感组；绿色：耐药组） Specific peptide peaks of model (red: sensitive group Ⅰ; green: resistant group Ⅰ)

### 2.5 盲法验证

验证组通过同样方法进行质谱检测，并对所建立的疗效预测模型进行盲样验证。9例化疗敏感型患者（9/10）和8例耐药型患者（8/10）被准确诊断。10例敏感型患者中有1例假阴性，10例耐药型患者中有2例假阳性。该模型的准确率为85.0%，灵敏度为90.0%，特异性为80.0%。验证结果见表 3。

**3:** 验证组结果 Result of validation group

Prognosis model	Chemotherapy response		
	Good	Bad	Total
Good	9	2	11
Bad	2	8	10
Total	11	9	20

## 3 讨论

SCC是肺癌中常见的病理类型，约占肺癌新发病例的1/4^[15, 16]^。近几年来，靶向治疗、免疫治疗及抗血管生成治疗等各种治疗方式进展迅速，许多新药已进入临床实践阶段^[17-20]^。但晚期SCC一线治疗仍然主要以化疗为主，其标准铂二联方案化疗只能给患者带来有限的获益^[3]^。其总缓解率约为25%-35%，中位PFS 4个月-6个月，OS为8个月-10个月^[21]^。并且不同的患者对于化疗药物的获益不同。所以实现化疗药物最优选择达到个体化预见性治疗尤为重要。近年来虽然进行了多项针对单基因标志物如ERCC1、RRM1、TUBB3及XRCC1的研究，并初步显示出一定意义，但在进一步扩大样本验证的随机对照Ⅲ期临床研究中均未能有效预测疗效^[4, 5]^。Bepler等^[4]^在晚期NSCLC患者中进行了*ERCC1*和*RRM1*基因表达与吉西他滨联合卡铂化疗疗效相关性的多中心Ⅲ期临床试验（NCT00499109），研究显示：实验组与对照组的PFS（6.1个月*vs* 6.9个月）及OS（11.0个月*vs* 11.3个月）均无统计学差异。由此可知单基因标志物对化疗疗效预测的意义非常有限。进一步的基础研究也表明细胞毒药物应答与单基因标志物的相关性较弱^[22, 23]^。已有研究表明药物代谢酶多态性可导致不同患者对于化疗药物的获益不同。因此通过蛋白质组学研究寻找到差异多肽可能会有效地预测化疗疗效。

近年来，蛋白质组学已广泛应用于肿瘤诊断、治疗等各方面的研究。其主要质谱检测技术包括表面加强激光解析电离飞行时间质谱（surface-enhanced laserdesorption ionization-time of flight-mass spectrometry, SELDI-TOFMS）、MALDI-TOF-MS等^[24]^。MOLID-TOF-MS技术为最具代表的蛋白质检测技术。MALDI技术是一种软电离技术，使生物大分子离子化，其具有灵敏度高、准确度高、稳定性好等特点^[25, 26]^，并被广泛应用于多种肿瘤的组织、血清及尿液蛋白质组学的研究中^[27, 28]^。Han等^[29]^运用SELDI-TOF-MS检测60例接受VP（依托泊苷+顺铂）方案化疗的广泛期小细胞肺癌（small cell lung cancer, SCLC）患者治疗前血清多肽。发现SCLC患者VP方案化疗耐药相关多肽并运用3个在耐药型患者血清样本中表达明显增高的差异多肽建立了耐药模型。但遗憾的是，后面研究者进行重复验证时并未得到一致结果。究其原因可能与SELDI-TOF-MS技术的耐污染物能力、稳定性、结果可靠性及重复性均较差有关^[30]^。我们的研究采用了稳定性较好的MALDI-TOF-MS检测初治晚期SCC患者接受紫杉醇类联合铂类化疗前血清多肽，并分析其与化疗疗效的相关性。检测化疗前血清样本发现化疗敏感型与耐药型患者间存在血清多肽差异。在800 Da-10, 000 Da范围内，检测到96个差异多肽，统计学处理后具有统计学意义（*P* < 0.001）的差异多肽有16个。运用1, 897.75 Da、2, 023.93 Da、3, 683.36 Da、4, 269.56 Da、5, 341.29 Da共5个多肽建立疗效预测模型。该模型对化疗敏感组患者的识别率为95.11%，交叉验证率为89.18%。经验证组验证其模型具有较高的灵敏度（90%）和特异性（80%），且表现出较好的稳定性及可重复性。

杨邵瑜等^[31]^应用MALDI-TOF-MS检测25例初治晚期NSCLC患者接受吉西他滨联合铂类方案化疗前血清多肽与化疗疗效的相关性。筛选出5个差异多肽在耐药组患者中表达明显增高，考虑可能与吉西他滨及顺铂耐药相关。但其仅检测到敏感组与耐药组患者血清多肽中存在差异，并未建立诊断模型进行化疗疗效的预测，也未分析差异多肽与临床预后参数之间的相关性。与其相比，本研究样本量较大，且全部样本均来自临床治疗以化疗为主的晚期SCC患者。不仅检测到了血清差异多肽，初步建立了疗效预测模型。而且更进一步对血清多肽与化疗疗效的相关性进行了分析。将具有统计学意义的差异多肽按照P值由低到高排列。结合临床预后参数PFS运用双变量相关分析，得到各个差异多肽与PFS间的相关系数。结果发现：m/z为4, 232.04 Da、4, 269.56 Da的差异多肽与SCC患者PFS存在相关性（*P* < 0.001）。该结果显示这2个多肽与SCC患者接受紫杉醇类联合铂类方案化疗的疗效密切相关，其相关性具有统计学意义。其中4, 269.56 Da的差异多肽峰同样用于建立疗效预测模型，进一步说明了其多肽与化疗疗效的密切关系。既往松本和子等^[32]^也通过检测接受曲妥珠单抗治疗的乳腺癌患者血浆中的糖酵解产物，找到R-L岩藻糖酶的表达与患者的PFS显著相关。并运用m/z为2, 534 Da的差异多肽对患者进行PD与非PD的分组，其灵敏度为75%，特异性为82%。研究显示乳腺癌患者血浆中存在与曲妥珠单抗敏感性明确相关的糖酵解生物标记物，可对接受曲妥珠单抗治疗的乳腺癌患者进行疗效预测。但与我们的研究相比，松本和子并未建立疗效预测的分类模型，而是通过单个差异多肽进行PD组与非PD组的区分，因此其灵敏度和特异性均较低。

本研究通过运用MALDI-TOF-MS技术对接受紫杉醇类联合铂类方案化疗的晚期SCC患者的血清样本进行了质谱检测，发现化疗敏感型与耐药型SCC患者间存在血清多肽差异。应用CPT软件建立了能够区分敏感型及耐药型SCC患者的分类模，型并进行了初步验证，得到了较稳定的结果。但本研究为单中心研究，样本量有限，下一步将扩大样本量完善所建立的分类模型，并对检测到的差异多肽进行进一步鉴定，以期找到能够预测化疗效果的血清标志物，最终实现肺癌的个体化化疗。
